# Accuracy of real-time PCR assays for human papillomavirus using urine samples: a systematic review and meta-analysis

**DOI:** 10.1128/jcm.01352-24

**Published:** 2025-03-31

**Authors:** Byeong-Min Park, Soohyun Kim, Jieun Choi, Yoonkyung Song, Seungman Park

**Affiliations:** 1 Department of Laboratory Medicine, National Cancer Center, Goyang-si, Gyeonggi-do, South Korea; 2 Department of Health Promotion, National Health Insurance Service Ilsan Hospital 65413, Goyang-si, Gyeonggi-do, South Korea; 3 Department of Laboratory Medicine, National Children’s Medical Center, Tashkent, Uzbekistan; St Jude Children's Research Hospital, Memphis, Tennessee, USA

**Keywords:** human papillomavirus, cervical cancer screening, real-time PCR, urine-based testing meta-analysis, diagnostic accuracy

## Abstract

This study aims to assess the diagnostic accuracy of real-time PCR assays for detecting human papillomavirus (HPV) in urine samples through a systematic review and meta-analysis. A comprehensive search of PubMed, Embase, and Springer databases (2014–2024) was conducted. Studies comparing urine-based HPV tests with cervical samples as the reference standard were included. Diagnostic accuracy measures such as sensitivity, specificity, diagnostic odds ratio (DOR), likelihood ratios (LR+ and LR−), percent agreement, and Cohen’s kappa were calculated. Heterogeneity was assessed using the Higgins’ I² index, and subgroup analyses were performed based on HPV test type and urine volume. The study revealed that 15 studies met the inclusion criteria, with pooled sensitivity of urine-based HPV tests at 0.82 (95% CI, 0.78–0.86), specificity at 0.91 (95% CI, 0.87–0.94), positive LR at 9.5 (95% CI, 6.3–14.3), negative LR at 0.19 (95% CI, 0.16–0.24), DOR at 49 (95% CI, 32–75), and the area under the curve at 0.92 (95% CI, 0.90–0.94), with significant heterogeneity observed (I² >50%), particularly in sensitivity and specificity, and subgroup analysis indicating that urine volumes ≤20 mL demonstrated higher sensitivity compared to those >20 mL, despite this finding being based on a limited number of studies. Results suggest that urine-based HPV testing shows strong diagnostic accuracy and could be a viable alternative to cervical swabs, with potential benefits for increasing screening accessibility, especially in areas with limited healthcare resources, despite some variability and limitations in the data.

**IMPORTANCE:**

This study is significant as it thoroughly evaluates the diagnostic accuracy of real-time PCR assays for human papillomavirus (HPV) detection in urine samples through a rigorous systematic review and meta-analysis. By integrating data from multiple databases and comparing urine-based HPV tests with the established cervical sample reference standard, the study provides valuable insights into the effectiveness and reliability of non-invasive HPV screening methods. The findings demonstrate that urine-based tests exhibit high sensitivity and specificity, offering a promising alternative to traditional cervical swabs. This advancement has significant implications for increasing accessibility to HPV screening, particularly in under-resourced settings, thereby potentially enhancing cervical cancer prevention efforts on a broader scale. The study not only fills a critical gap in HPV screening methodologies but also supports the development of more inclusive and practical public health strategies for combating cervical cancer.

## INTRODUCTION

Human papillomavirus (HPV) infection is the most significant risk factor for cervical cancer, and approximately 70% of cervical cancer cases are caused by HPV types 16 and 18 (1). Since cervical cancer can be effectively treated if detected early in precancerous lesions, regular screenings are essential for both prevention and timely intervention (2).

The participation rate for cervical cancer screening exhibits a statistically significant decline from developed nations to developing regions (3). For example, only around 20% of women in lower-income countries have undergone cervical cancer screening (4), in stark contrast to the more than 60% participation rate observed in high-income countries (5, 6). Numerous factors contribute to the low uptake of cervical cancer screening in underdeveloped nations, with one of the primary challenges being the scarcity of medical personnel (7). Paradoxically, even in developed countries with relatively ample healthcare resources, approximately 20%–30% of women fail to engage in cervical cancer screening programs (8).

In addition to improving screening rates, increasing HPV vaccination coverage is crucial for reducing cervical cancer incidence, particularly in developing countries (9). Highly effective vaccines, such as Gardasil and Cervarix, prevent infections caused by HPV types 16 and 18, which account for the majority of cervical cancer cases (10). However, barriers such as limited healthcare access and affordability have resulted in low vaccination rates in many low- and middle-income countries (11). Integrating HPV vaccination programs with existing cervical cancer screening initiatives could significantly enhance prevention efforts and reduce the global burden of cervical cancer (12).

Although cervical swab samples are the standard method for HPV testing, studies on urine-based HPV tests have been increasingly reported due to advantages such as non-invasive techniques and the development of new test methods for convenience sampling (13, 14). Commercial real-time polymerase chain reaction (PCR) assays provide the advantage of automated batch-mode testing (15), providing faster test results than Pap smears (16, 17). Since the results are presented as quantitative outputs, such as cycle threshold values or viral copy numbers, they enable objective interpretation and facilitate standardized monitoring over time (18). Therefore, this study aims to evaluate the diagnostic accuracy of the real-time PCR assays for HPV detection in urine samples via a systematic review and meta-analysis.

## MATERIALS AND METHODS

### Literature search and criteria for selection/exclusion

**Fig 1:**
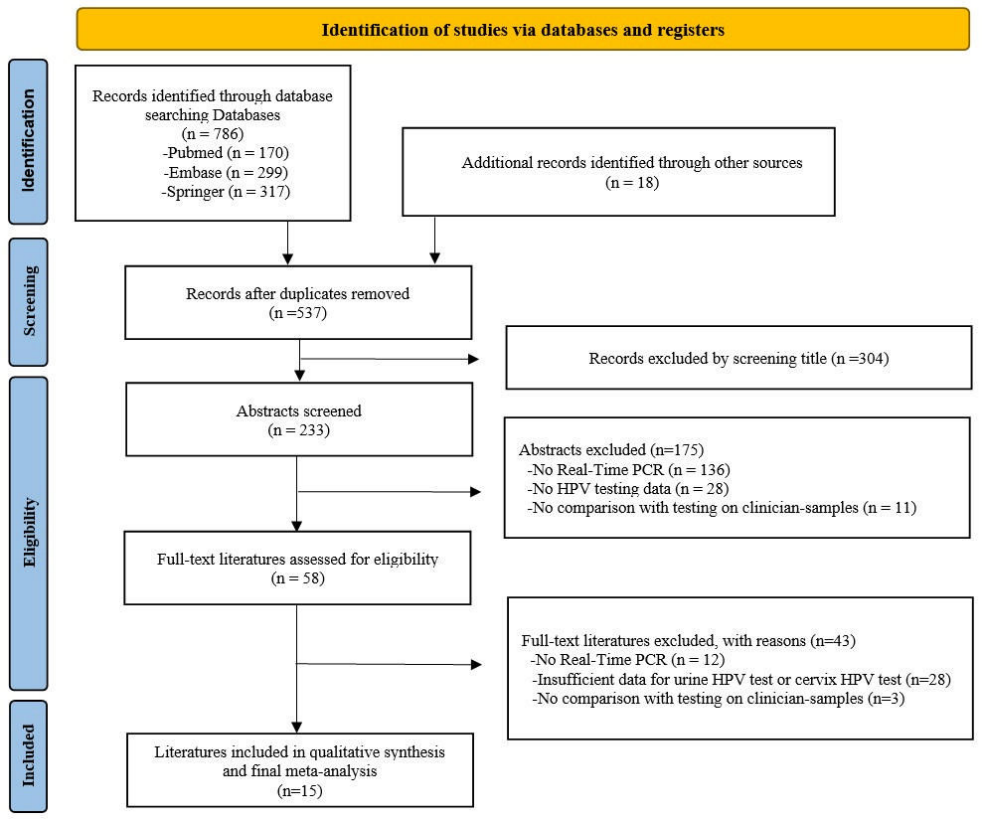
Flowchart illustrating the study selection process for eligible studies.

A comprehensive search of electronic databases including PubMed, Embase, and Springer was conducted, covering publications from 2014 to 2024. Key search terms such as “HPV,” “human papillomavirus,” “cervical cancer screening,” “real-time PCR,” and “urine” were employed during the database search. Articles were excluded if they were duplicates, were unrelated, or lacked sufficient data (Fig. 1). Studies utilizing non-real-time PCR methods, such as transcription-mediated amplification (TMA)-based assays (e.g., Aptima HPV test) (19), were intentionally excluded to ensure methodological consistency and minimize heterogeneity in the pooled analysis. Although TMA is a reliable diagnostic tool for detecting HPV, its amplification method fundamentally differs from real-time PCR. This difference causes variations in interpretation, making direct comparisons difficult. Cervical samples were used as the reference standard, while urine samples served as the index test to construct 2 × 2 contingency tables for data analysis. In all 15 included studies, both cervical and urine samples were analyzed using the same real-time PCR assays, ensuring methodological consistency across sample types. This approach enabled a direct and reliable comparison of HPV detection performance between cervical and urine samples. A summary of the qualitative characteristics of the selected studies is provided in Table 1.

**TABLE 1:** Qualitative characteristics of selected studies^*a*^

Year	Author (Ref)	TP	FP	FN	TN	Sample size	Population	Prevalence %	HPV detection assays	Sample preparation	Timing	Urine volume	Age (range)
2014	Bernal et al. (20)	57	9	6	53	125	Retrospective	50.4	Cobas 4800 HPV test	Pellet	First-voided urine	20 mL	Median 35.5 (21–65)
2015	Stanczuk et al. (21)	78	1	14	7	100	Retrospective	92.0	Cobas 4800 HPV test	Pellet	N/A	20 mL	Median 27.5 (21–60)
2016	Hagihara et al. (22)	98	5	22	115	240	General	50.0	Anyplex II HPV 28 detection kit	Pellet	First-voided urine	N/A	Median 31 (19–58)
2016	Khunamornpong et al. (23)	24	6	11	82	123	Retrospective	28.5	Cobas 4800 HPV test	Pellet	First-voided urine	5 mL–50 mL	Mean 45.8 (± SD 8.1)
2017	Asciutto et al. (24)	141	27	8	39	215	Retrospective	69.3	Cobas 4800 HPV test	Unprocessed	First-voided urine	N/A	Mean 35.2 (19–71)
2017	Lim et al. (25)	57	3	21	119	200	Retrospective	39.0	Cobas 4800 HPV test	Pellet	First-voided, mid-stream urine	30 mL	N/A
2017	Lim et al. (25)	55	4	21	120	200	Retrospective	38.0	RealTime High Risk HPV test	Pellet	First-voided, mid-stream urine	30 mL	N/A
2018	Vergara et al. (26)	289	12	63	179	543	General	64.8	Non-commercial software	Pellet	First-voided urine	10 mL–15 mL	Mean 36 ± 11.1 (18–64)
2019	Sargent et al. (27)	45	3	11	20	79	Retrospective	70.9	RealTime High Risk HPV test	Unprocessed	First-voided urine	N/A	40% under 30 years
2019	Sargent et al. (27)	44	1	4	17	66	Retrospective	72.7	Cobas 4800 HPV test	Unprocessed	First-voided urine	N/A	N/A
2020	Tranberg et al. (28)	23	4	13	110	150	Retrospective	24.0	Cobas 4800 HPV test	Pellet	First-voided urine	10 mL–12 mL	30–59
2021	Cho et al. (29)	194	16	53	51	314	Retrospective	78.7	RealTime High Risk HPV test	Pellet	First-voided urine	30 mL	20–60
2021	Cho et al. (29)	164	13	66	71	314	Retrospective	73.2	Anyple II HPV28 assay	Pellet	First-voided urine	30 mL	20–60
2021	Van Keer et al. (30)	247	36	46	164	493	Retrospective	59.4	RealTime High Risk HPV test	Unprocessed	First-voided urine	13 mL	Median 40 (19–72)
2022	Kim et al. (31)	19	7	7	177	210	General	12.4	PANA RealTyper HPV Screening Kit using CFX96	Pellet	Random voided urine	N/A	Mean 39.9 (20–85)
2022	Punyashthira et al. (32)	66	4	12	14	96	Retrospective	81.2	Anyplex II HPV high-risk	Unprocessed	First and random voided urine	10 mL–15 mL	Mean 47.5
2023	Martinelli et al. (33)	149	12	11	73	245	Retrospective	65.3	Anyplex II HPV28 assay	Unprocessed	N/A	20 mL	Mean 38.6 (17–67)
2024	Nilyanimit et al. (34)	34	4	4	198	240	General	15.8	Cobas 4800 HPV test	Pellet	First-voided urine	20 mL	Mean 46 (22–70)

^*a*^ TP, true positive; FP, false positive; FN, false negative; TN, true negative; N/A, not available.

### Quality assessment

The quality of the studies was assessed by two independent reviewers (B.M. and S.H.) using the Quality Assessment of Diagnostic Accuracy Studies 2 (QUADAS-2) tool (35). This QUADAS-2 tool comprises four key domains: patient selection, index test, reference standard, and flow and timing. Each domain includes a systematic evaluation of both bias risk and concerns regarding applicability. For each detailed item within each domain, bias risk is categorized as either “yes,” “no,” or “unclear,” while overall bias risk and applicability concerns are rated as “low,” “high,” or “unclear” for each domain. In cases of discordant evaluations, consensus was achieved through discussions between the reviewers.

**Fig 2:**
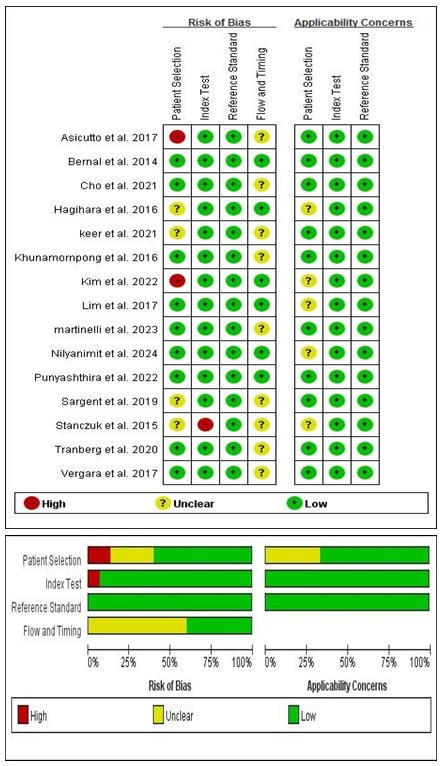
Quality assessment of the 15 included studies using QUADAS-2.

All 15 included studies underwent quality assessment using the QUADAS-2, and no studies were excluded based on this assessment. The QUADAS-2 effectively identified potential biases across the included studies (36). Six studies lacked clarity in reporting patient selection, one study lacked clarity in reporting the index test, and nine studies lacked clarity in reporting flow and timing. Concerns regarding applicability were also flagged in five studies due to incomplete reporting of patient selection. These limitations were carefully considered during data synthesis and interpretation to ensure a balanced evaluation of the findings. Despite these flagged concerns, the overall reliability of the meta-analysis findings was supported by consistent diagnostic performance across studies, as reflected by high pooled sensitivity and specificity values (37). However, these flagged limitations underscore the importance of cautious interpretation in specific subgroups or contexts where generalizability may be limited. Figure 2 illustrates the quality assessment of the 15 included studies using the QUADAS-2.

### Statistical analysis

The primary objective was to summarize the overall diagnostic performance (38, 39). Diagnostic performance was evaluated in terms of sensitivity, specificity, diagnostic odds ratio (DOR), positive likelihood ratio (LR+), negative likelihood ratio (LR−), percent agreement, and Cohen’s kappa, with results presented along with 95% confidence intervals (CIs). A bivariate random effects model was used to simultaneously consider sensitivity and specificity, and a summary receiver operating characteristic (SROC) curve was analyzed (40, 41). Results from different HPV detection tests within a single study were treated as separate studies. A forest plot was used to visually present the pooled diagnostic performance results. The Higgins’ inconsistency index (I^2^) was calculated to assess heterogeneity among studies (42), and subgroup differences were tested using a random effects model to determine whether differences in HPV detection tests and urine volumes contributed to this heterogeneity. HPV detection tests were classified into four groups: Cobas 4800, Anyplex II, RealTime High-Risk, and others. Urine volume was classified as ≤20 mL and >20 mL, and studies that did not report values or could not be classified were treated as missing. Publication bias was assessed using Deeks’ funnel plot, which was developed to examine diagnostic performance (43). All statistical analyses were conducted using statistical software, including Stata version 12.0 (StataCorp, College Station, TX, USA) and R version 4.1.1 (R Foundation for Statistical Computing, Vienna, Austria), with statistical significance defined as *P* < 0.05.

## RESULTS

### Study selection and characteristics

A total of 804 articles were identified, 786 from primary databases (PubMed, Embase, and Springer) and 18 additional articles through other sources, including manual reference list searches and expert recommendations. After applying the inclusion and exclusion criteria, the final data set of 15 studies was derived exclusively from primary database searches, as none of the articles from other sources met the eligibility criteria. After removing duplicates, 537 records remained. These were systematically narrowed down through title and abstract screening, which excluded 479 records based on relevance and specific eligibility criteria. A subsequent full-text assessment further refined the selection, eliminating 43 studies that did not fully meet the inclusion criteria. Ultimately, 15 studies were chosen for qualitative synthesis and meta-analysis, forming the foundation of the review outcomes (20–34). Figure 1 illustrates the flow of the database search and literature selection process. Table 1 provides a summary of the selected studies’ characteristics, including year of publication, author, true positive, false positive, false negative, true negative, sample size, population prevalence, HPV detection assays, sample preparation methods, urine volume, and participant age range. The risk of bias assessment identified concerns in patient selection, index test, reference standard, and flow and timing. Specifically, six studies lacked clarity in reporting patient selection, one study lacked clarity in reporting the index test, none of the studies lacked clarity in reporting the reference standard, and nine studies lacked clarity in reporting flow and timing. The assessment of concerns regarding applicability indicated that the study results might not be applicable to clinical practice situations in some cases, such as the five studies with incomplete reporting of patient selection (Fig. 2).

### Diagnostic performance and agreement in urine samples compared to cervical samples

**Fig 3:**
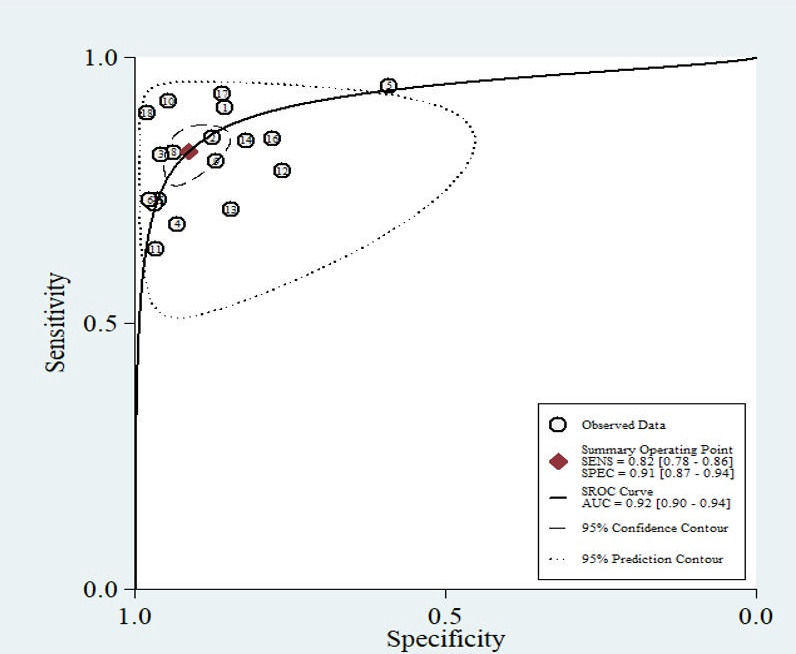
SROC curve with 95% confidence and prediction regions around the mean operating sensitivity and specificity point.

**Fig 4:**
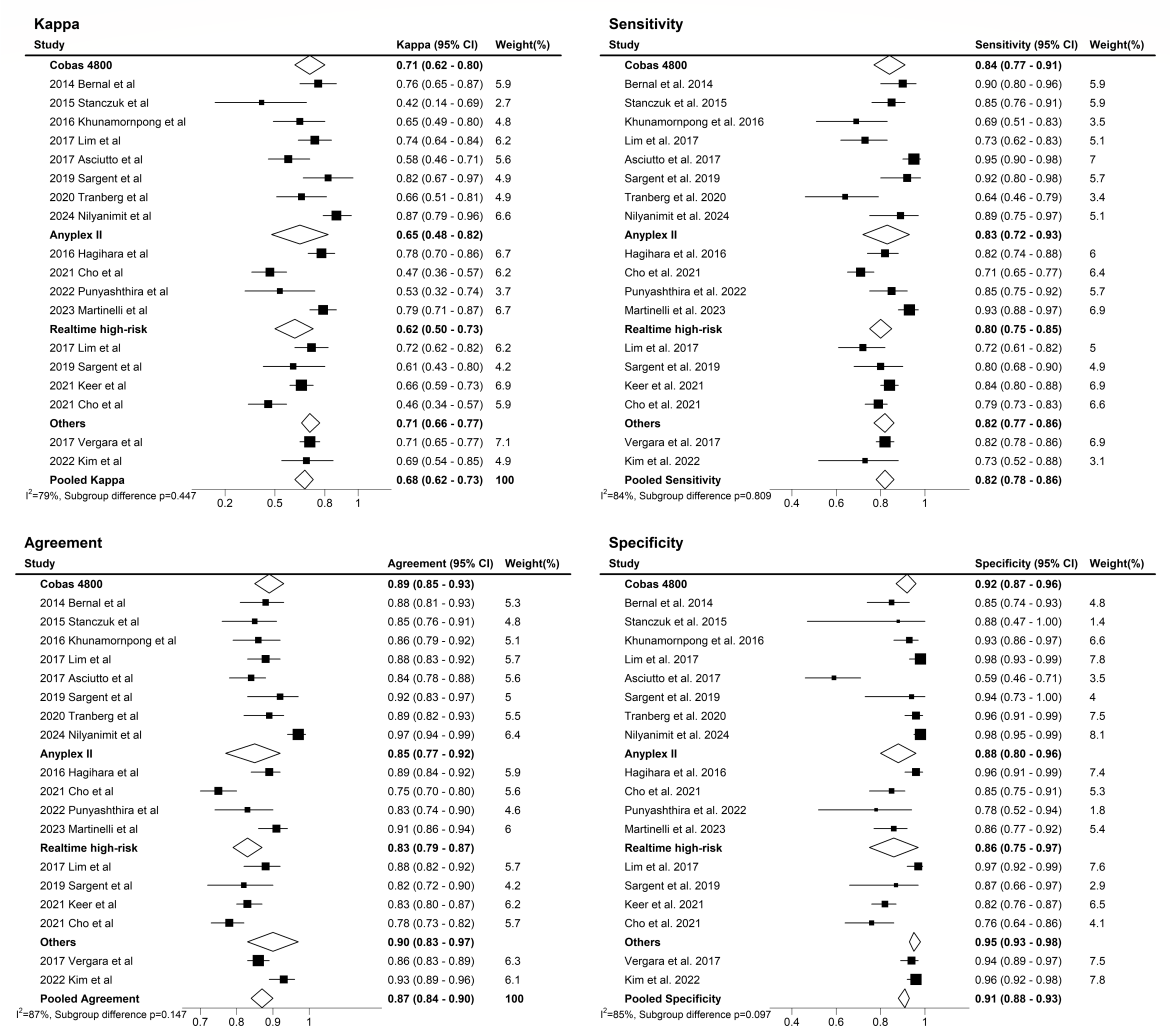
Meta-analysis assessing heterogeneity in HPV detection performance and agreement across commercial real-time PCR systems in urine.

The HPV real-time PCR assays using urine samples demonstrated a pooled sensitivity of 0.82 (95% CI, 0.78–0.86), specificity of 0.91 (95% CI, 0.87–0.94), LR+ of 9.5 (95% CI, 6.3–14.3), LR− of 0.19 (95% CI, 0.16–0.24), and diagnostic odds ratio of 49 (95% CI, 32–75) (Table 2). The area under the curve (AUC) of the SROC curve was 0.92 (95% CI, 0.90–0.94), and as a result of visually inspecting the heterogeneity of the SROC curve, the symmetry of the curve and the spread of individual studies were appropriate, leading to no suspicion of heterogeneity among the included studies (Fig. 3). The pooled kappa was 0.68 (95% CI, 0.62–0.73), and the percent agreement was 0.87 (95% CI, 0.84–0.90) (Fig. 4).

**TABLE 2:** Summary of diagnostic performance for HPV testing on urine in the 15 studies reviewed^*a*^

Parameter	Estimate	95% CI
Sensitivity	0.82	0.78–0.86
Specificity	0.91	0.87–0.94
Positive likelihood ratio	9.5	6.3–14.3
Negative likelihood ratio	0.19	0.16–0.24
Diagnostic odds ratio	49	32–75

^*a*^ 95% CI, 95% confidence interval.

**Fig 5:**
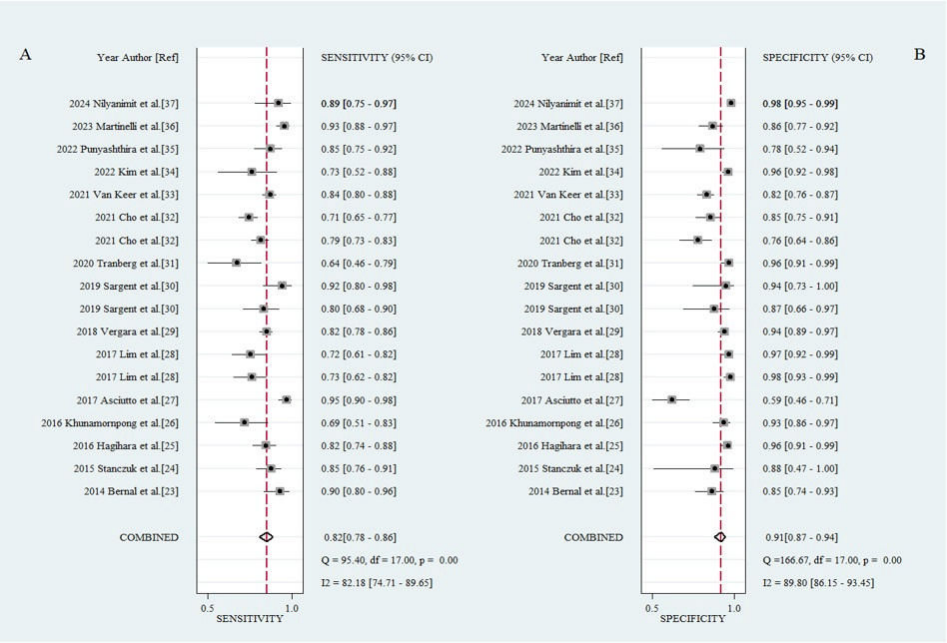
Forest plot illustrating (A) sensitivity and (B) specificity of real-time PCR assays for HPV detection in urine samples.

### Heterogeneity, meta-regression, and publication bias

The Higgins’ I^2^ index, used to quantify the degree of heterogeneity in a meta-analysis (42), showed values of 82.2% for sensitivity, 89.8% for specificity, 78.8% for kappa, and 87.2% for percent agreement (Fig. 4 and 5). I^2^ values greater than 50% were considered to indicate substantial heterogeneity. In cases of significant heterogeneity in meta-analysis, it is possible to explore the causes of heterogeneity (44). Accordingly, the subgroup analyses using a random effects model were conducted to assess the impact of HPV detection test type and urine volume based on the study characteristics.

**Fig 6:**
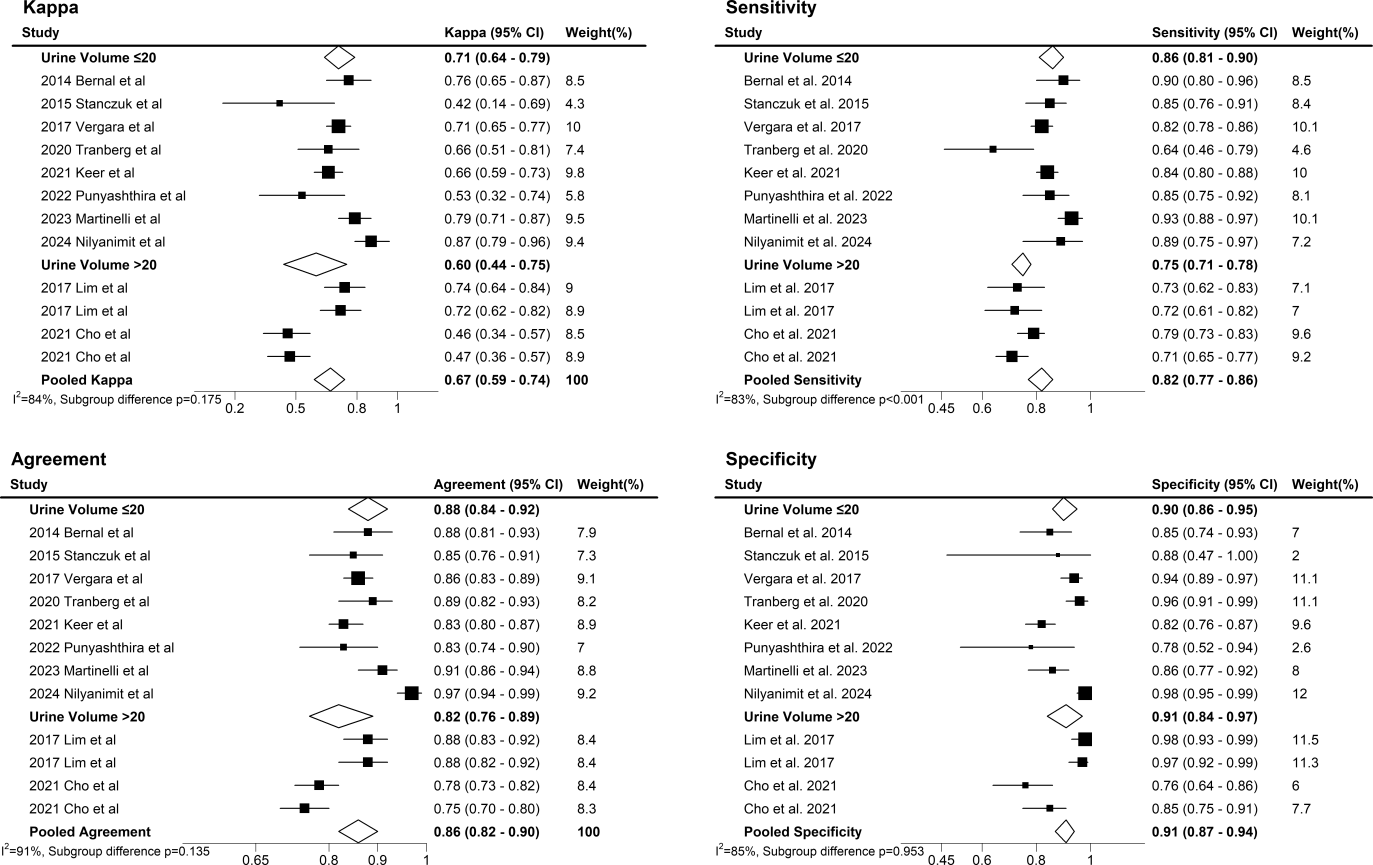
Effect of urine volume thresholds (≤20 mL vs >20 mL) on HPV concordance and heterogeneity in the meta-analysis.

In the HPV detection test subgroup, the subgroup difference test *P-*values for sensitivity, specificity, kappa, and percent agreement were 0.809, 0.097, 0.447, and 0.147, respectively, indicating no statistically significant difference. Although no statistically significant differences were observed in sensitivity and specificity, the Cobas 4800 test exhibited numerically higher diagnostic values, indicating potential clinical significance (Fig. 4). Further subgroup analysis based on urine volume showed no significant differences in specificity, kappa, and percent agreement, with *P-*values of 0.953, 0.175, and 0.135, respectively. However, a statistically significant difference in sensitivity was observed between urine volume subgroups (0.857 vs 0.746, *P* < 0.001), with higher sensitivity observed in samples with low urine volume (Fig. 6).

**Fig 7:**
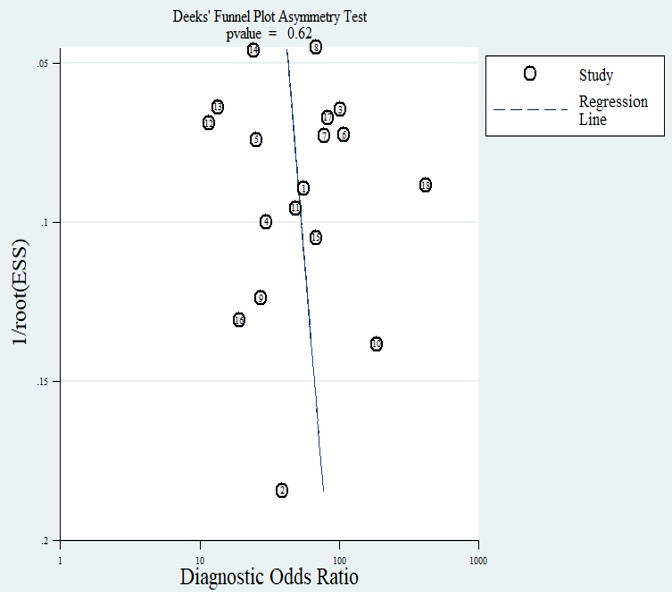
Deeks’ funnel plot for assessing publication bias.

A funnel plot was employed to assess the presence of publication bias in the reviews (45). Deeks’ test for publication bias (43) yielded a *P-*value of 0.62, indicating a low likelihood of publication bias in this meta-analysis (Fig. 7).

## DISCUSSION

HPV is spread through sexual contact, and approximately 311,000 women died from cervical cancer in 2018 (46), with more than 85% of these deaths occurring in low- and middle-income countries (47), underscoring the urgent need for improved screening strategies. Numerous studies have been conducted to support women in medically vulnerable groups, particularly in cervical cancer screening (48). Among the promising alternatives, urine specimens have been widely studied as potential replacements for cervical samples (49, 50). Urine samples are particularly attractive due to their ease of collection in a non-invasive manner. Notably, first-void urine samples have demonstrated higher detection rates of high-risk HPV (51); however, they may be susceptible to vulvar contamination, which could limit their accuracy in detecting cervical HPV infection (52). This limitation must be considered when interpreting urine-based HPV testing results and highlights the need for further research to clarify the exact relationship between HPV detection in urine and cervical infection. Despite the potential advantages of urine-based testing, the current standard screening methods are the Pap test and the HPV test (53). These tests are considered the gold standard for detecting cervical cell abnormalities and HPV presence. Generally, in meta-analyses, the sensitivity and specificity of the HPV test using DNA PCR with liquid-based cytology have been found to be higher than the Pap test, which involves sampling cells directly from the cervix (54).

In this study, HPV real-time PCR assays using urine samples demonstrated a pooled sensitivity of 0.82 (95% CI, 0.78–0.86) and a specificity of 0.91 (95% CI, 0.87–0.94), indicating a high concordance between urine and cervical samples for HPV detection, while suggesting potential discrepancies that require further investigation. Additionally, to establish the diagnostic utility of a general screening test, the combined value of sensitivity and specificity should be at least 1.5 (150%), a threshold referenced in evidence-based literature, as a practical benchmark for balancing sensitivity and specificity, minimizing false positives and negatives, and ensuring reliable diagnostic performance (55, 56).

However, the pooled specificity of 0.91 suggests that HPV was detected in urine samples but not in cervical samples, indicating potential false positives (57). Most studies included in this meta-analysis did not conduct comprehensive discrepancy analyses to confirm these results. Future research should incorporate rigorous discrepancy resolution protocols, such as repeat testing, longitudinal follow-up, and co-testing with Pap smears, to gain a more accurate understanding of urine-based HPV testing.

In this study, the agreement between tests, as indicated by the percent agreement and kappa, was measured at 0.87 (95% CI, 0.84–0.90) and 0.68 (95% CI, 0.62–0.73), respectively. When assessing the consistency between tests, particularly with categorical data, researchers commonly employ statistical measures such as kappa or percent agreement (58). Generally, inter-rater reliability data for categorical variables are considered inadequate if percent agreement falls below 80% or if kappa is 0.6 or lower (59). The data were reviewed again due to a few studies reporting low kappa values; however, the results remained consistent. Notably, in the study by Stanczuk et al., the gold standard exhibited a distribution of 92% HPV-positive versus 8% HPV-negative cases. This uneven distribution likely contributed to the lower calculated kappa value (60, 61). These observations clearly highlight the inherent limitations of using kappa. Specifically, a notable limitation of kappa is its sensitivity to variations in evaluator assignment, regardless of agreement (62). The degree of homogeneity in the data distribution can significantly influence kappa values, with greater uniformity leading to higher kappa values, and when marginal homogeneity is satisfied (i.e., when off-diagonal cells are equal, A1 = B1), resulting in lower kappa values. In essence, the observed percent agreement may remain consistent in this study, while kappa can vary substantially depending on the data distribution. Although slightly below the commonly accepted threshold of 90% (63), we propose that percent agreement remains a more suitable measure for this study, given its relatively high overall value (0.87) and reduced susceptibility to prevalence-related bias observed in kappa values from skewed data distributions (64).

The Higgins’ I^2^ values range from 0 to 100% and represent the proportion of inter-study variability as heterogeneity. Values of 25%, 50%, and 75% are interpreted as indicating low, moderate, and high estimates of heterogeneity, respectively (65). In this study, I² values of 82.18 for sensitivity and 89.80 for specificity indicate high heterogeneity according to these criteria. The included studies demonstrated variability in the sample preparation methods, with most utilizing centrifugation to concentrate urine and analyze the pellet. Some studies used unprocessed samples, and none independently tested the supernatant. This variability likely influenced assay sensitivity, as previous research has shown that preparation methods such as centrifugation can significantly increase HPV DNA concentration (66), thereby enhancing diagnostic accuracy. As shown in Table 1, prevalence rates ranged from 12.4% to 92.0%, with retrospective studies reporting higher rates due to selection biases in high-risk populations, whereas prospective studies targeting general populations reported lower prevalence (67). These differences, along with variability in sample preparation methods, urine volumes, and study population characteristics (51), substantially contributed to the observed heterogeneity (sensitivity: I² = 82.18%; specificity: I² = 89.80%; Fig. 5) in this meta-analysis. Rigorous protocols, including repeat testing, longitudinal follow-up, and co-testing with Pap smears, have been suggested in previous literature to improve diagnostic accuracy and address potential discrepancies (68). Future research should focus on the standardization of preparation methods, stratifying results by factors such as vaccination status and screening history, and prioritizing general screening populations to enhance the generalizability and comparability of findings. To this end, it is important to identify the causes of heterogeneity and conduct subgroup and sensitivity analyses (69, 70). Moreover, diagnostic tests commonly exhibit inherent variability due to factors such as diverse patient populations, variations in techniques, and other contributing factors (71, 72).

Previous studies have indicated that there is no statistically significant difference in HPV detection rates for urine volumes below 20 mL, and that first-void urine is more suitable than midstream urine (51, 73). In this meta-analysis, samples with urine volumes below 20 mL demonstrated significantly higher sensitivity compared to those with volumes exceeding 20 mL. This suggests that dilution effects may reduce the sensitivity of assays when larger urine volumes are used. In this meta-analysis, most of the included studies employed centrifugation to concentrate urine and analyze the pellet, a method known to enhance HPV DNA detection rates by increasing its concentration (74). However, as summarized in Table 1, only a few studies analyzed unprocessed urine samples, and none examined the supernatant independently. A limitation of this meta-analysis is that the fraction of urine analyzed was often unclear or randomly collected. Consequently, due to the limited number of studies that exclusively analyzed first-void urine, we included results from different sampling times when comparing urine volumes. Further studies are required to systematically evaluate the diagnostic impact of dilution and concentration methods.

The pooled sensitivity of 82% (95% CI, 0.78–0.86) for urine-based HPV testing, although lower than those of cervical sampling methods, should be interpreted in the context of its high specificity of 91% (95% CI, 0.87–0.94) and its potential to improve screening accessibility (Table 2; Fig. 5) (51). Subgroup analysis revealed that urine volumes ≤20 mL demonstrated significantly higher sensitivity compared to volumes >20 mL (0.857 vs 0.746, *P* < 0.001; Fig. 6). This underscores the critical importance of standardized urine collection protocols in optimizing diagnostic accuracy (75). While a detailed discrepancy analysis of false-positive results was not feasible in this meta-analysis due to data limitations, potential sources of false positives, including contamination and assay variability, have been discussed in previous studies (76). Urine-based testing offers a promising complementary approach for HPV screening, particularly in settings with limited healthcare resources. Future studies should prioritize standardizing urine collection methods, improving assay specificity, and conducting large-scale prospective studies to validate the diagnostic performance of urine-based HPV testing (77).

Another limitation of this study is that the included studies did not consistently differentiate between screening-naïve versus previously screened populations or vaccinated versus unvaccinated individuals. Previous research has shown that vaccinated individuals typically exhibit lower HPV prevalence and are less likely to test positive for HPV (78), while individuals with regular screening are more likely to identify cervical abnormalities at earlier stages, leading to improved diagnostic outcomes (79). The absence of stratification for these critical factors is likely to have contributed to variability in the pooled diagnostic accuracy. Future studies should systematically report these variables to enable comprehensive subgroup analyses and improve our understanding of HPV test performance across diverse populations.

In other research findings, meta-regression identified the first urine as a statistically significant source of heterogeneity, showing higher levels of high-risk HPV compared to other urine fractions (80, 81). Another study corroborated its potential as a urinary biomarker for cervical cancer screening (75). In this study, the pooled kappa and agreement for heterogeneity analysis of commercial PCR systems for HPV detection were highest for the Roche Cobas 4800, followed by the Seegene Anyplex II HPV28 detection and Anyplex II HPV HR detection, and then the Abbott RealTime High-Risk HPV. However, the test *P-*values comparing differences between subgroups, including those for non-commercial PCR systems, did not show statistically significant differences. Given that a limited number of studies can lead to inaccuracies in estimates of heterogeneity (82), it will be essential to collect more data to accurately assess and understand the underlying causes of heterogeneity in future investigations.

The LR indicates how much more likely a test result is to occur in patients with the disease compared to those without it (83). Likelihood ratios calculated as integrated estimates of sensitivity and specificity reveal crucial diagnostic insights (84). An LR of 1 indicates that the test is not diagnostically useful, while LR values above 10 for positive LR and below 0.1 for negative LR signify a test that is definitely clinically useful, with the potential to alter clinical decisions. Positive LR values above 5 and negative LR values below 0.2 provide strong diagnostic evidence (85). In this study, urine-based HPV testing demonstrated a positive LR of 9.5 (95% CI, 6.3–14.3) and a negative LR of 0.19 (95% CI, 0.16–0.24).

The DOR is another measure of diagnostic accuracy that is calculated by combining the likelihood ratios (LR+ and LR−). It ranges from 0 to infinity, with higher values indicating better discriminatory test performance (86). Tests with DOR values exceeding 20 are generally considered potentially useful, although this threshold can vary depending on the test’s sensitivity and specificity (87). In other words, as sensitivity and specificity increase and false positive and false negative rates decrease, the DOR is likely to increase. In this study, the DOR value was calculated at 49 (95% CI, 32–75), demonstrating good diagnostic accuracy.

ROC analysis is a valuable tool for assessing the performance of diagnostic tests, and the ROC curve illustrates the trade-off between sensitivity and specificity calculated based on varying cutoff values of test results measured on a continuous scale (88). The AUC, which represents the area under the ROC curve, serves as an overall summary of diagnostic accuracy, with a value of 1 indicating a perfect diagnostic test and a value below 0.5 suggesting the test is ineffective (89). In this meta-analysis, the AUC was calculated to be 0.92, indicating strong diagnostic performance for urine-based HPV detection.

Cervical cancer screening rates remain suboptimal worldwide due to various barriers. Limited healthcare access is a major factor in resource-constrained settings, but cultural beliefs, feelings of shame, and the stigma surrounding cervical examinations act as significant deterrents across diverse populations (90, 91). These sociocultural factors often discourage women from undergoing traditional Pap smears. Non-invasive alternatives, such as urine-based HPV testing, offer a promising solution by eliminating invasive procedures, reducing stigma, and enabling private sample collection (92, 93). Our meta-analysis highlights the reliable diagnostic accuracy of urine-based HPV testing (pooled sensitivity: 0.82, 95% CI: 0.78–0.86; specificity: 0.91, 95% CI: 0.87–0.94) (Table 2). This suggests that urine-based HPV testing could serve as a viable alternative to cervical swabs. By addressing cultural barriers and prioritizing patient comfort, these approaches can encourage greater participation in screening programs and promote early diagnosis worldwide.

### Conclusion

Urine-based HPV detection may be less effective than cervical sampling due to the etiology of cervical cancer. However, the high sensitivity, specificity, and strong concordance with cervical samples suggest that urine-based real-time PCR assays offer a viable, non-invasive alternative. These findings support the potential role of urine-based HPV testing in expanding screening accessibility, particularly in settings with limited healthcare resources, ultimately contributing to improved public health outcomes.
